# Splenic Marginal Zone Lymphoma in Turkey: Association with Hepatitis B Instead of Hepatitis C Virus as an Etiologic and Possible Prognostic Factor - A Multicenter Cohort Study

**DOI:** 10.4274/tjh.galenos.2019.2019.0177

**Published:** 2020-05-06

**Authors:** Müfide Okay, Tuncay Aslan, Evren Özdemir, Ayşegül Üner, Arzu Sağlam, Elif Güngör, Ayşe Uysal, Nevin Alayvaz Aslan, Esra Yıldızhan, Abdullah Ağıt, Mehmet Sinan Dal, Serdal Korkmaz, Sinem Namdaroğlu, Serdar Sivgin, Gülsüm Akgün Çağlıyan, Sinan Demircioğlu, İbrahim Barışta, Esra Özhamam, Filiz Vural, Bülent Eser, Gülsüm Özet, Rahşan Yıldırım, Mehmet Hilmi Doğu, İlhami Berber, Mehmet Ali Erkurt, Ümit Yavuz Malkan, Fevzi Altuntaş, Yahya Büyükaşık

**Affiliations:** 1 Hacettepe University Faculty of Medicine, Department of Internal Medicine, Division of Hematology, Ankara, Turkey; 2 Medicana International Ankara Hospital, Clinic of Medical Oncology, Ankara, Turkey; 3 Hacettepe University Faculty of Medicine, Department of Pathology, Ankara, Turkey; 4 Trakya University Faculty of Medicine, Department of Internal Medicine, Edirne, Turkey; 5 University of Health Sciences, Trabzon Kanuni Training and Research Hospital, Division of Hematology, Trabzon, Turkey; 6 Ondokuz Mayıs University Faculty of Medicine, Department of Hematology, Samsun, Turkey; 7 Erciyes University Faculty of Medicine, Department of Hematology, Kayseri, Turkey; 8 Ankara Numune Training and Research Hospital, Division of Hematology, Ankara, Turkey; 9 University of Health Sciences, Ankara Oncology Training and Research Hospital, Clinic of Hematology and BMT Unit, Ankara, Turkey; 10 Kayseri Training and Research Hospital, Division of Hematology, Kayseri, Turkey; 11 University of Health Sciences, İzmir, Turkey; 12 Acıbadem Kayseri Hospital, Kayseri, Turkey; 13 Denizli State Hospital, Division of Hematology, Denizli, Turkey; 14 Yüzüncü Yıl University Faculty of Medicine, Department of Hematology, Van, Turkey; 15 Hacettepe University Faculty of Medicine, Department of Medical Oncology, Ankara, Turkey; 16 Ankara Numune Training and Research Hospital, Division of Pathology, Ankara, Turkey; 17 Ege University Faculty of Medicine, Department of Internal Medicine, Division of Hematology, İzmir, Turkey; 18 Atatürk University Faculty of Medicine, Department of Hematology, Erzurum, Turkey; 19 İstanbul Training and Research Hospital, Clinic Hematology, İstanbul, Turkey; 20 Malatya Training and Research Hospital, Division of Hematology, Malatya, Turkey; 21 İnönü University Faculty of Medicine, Department of Internal Medicine, Division of Hematology, Malatya, Turkey; 22 University of Health Sciences, Dışkapı Yıldırım Beyazıt Training and Research Hospital, Clinic of Hematology, Ankara, Turkey; 23 Yıldırım Beyazıt University Faculty of Medicine, Department of Internal Medicine, Division of Hematology, Ankara, Turkey

**Keywords:** Low-grade lymphoma, Hepatitis B virus, Hepatitis C virus, Risk factors

## Abstract

**Objective:**

Chronic antigenic stimulation is frequently blamed in the pathogenesis of extranodal marginal zone lymphomas including splenic marginal zone lymphoma (SMZL). Chronic hepatitis C is frequently observed in SMZL patients in some geographical regions. However, these reports are largely from North America and Europe, and data from other countries are insufficient. In this multicenter study we aimed to identify the clinical characteristics of SMZL patients in Turkey, including viral hepatitis status and treatment details.

**Materials and Methods:**

Data were gathered from participating centers from different regions of Turkey using IBM SPSS Statistics 23 for Windows. Hepatitis B virus surface antigen (HBsAg), anti-HBs antibody, anti-HB core antigen antibody (anti-HBcAg), HB viral load, anti-hepatitis C virus (HCV) antibody, HCV viral load results were analyzed.

**Results:**

One hundred and four patients were reported. Hepatitis C virus positivity was observed in only one patient. However, hepatitis B virus surface antigen (HBsAg) positivity was observed in 11.2% and HBsAg and/or anti-HB core antigen antibody (anti-HBcAg) positivities were seen in 34.2% of the patients. The median age was 60 years (range=35-87). Median follow-up duration was 21.2 months (range=00.2-212; 23.2 months for surviving patients). Median overall survival was not reached. Estimated 3-year and 10-year survival rates were 84.8% and 68.9%, respectively. Older age, no splenectomy during follow-up, platelet count of <90x10^3^/μL, lower albumin, higher lactate dehydrogenase, higher β_2_-microglobulin, and HBsAg positivity were associated with increased risk of death. Only albumin remained significant in multivariable analysis.

**Conclusion:**

These results indicate that hepatitis B virus may be a possible risk factor for SMZL in our population. It may also be an indirect prognostic factor.

## Abstract (tr)

**Amaç:**

Splenik marjinal zon lenfoma’yı da (SMZL) içeren ekstranodal marjinal zon lenfomaların patogenezinde, kronik antijenik stimülasyon sorumlu olan mekanizmadır. Bazı coğrafik bölgelerde SMZL hastalarında kronik hepatit C sıklıkla gözlenir. Fakat bu çalışmalar sıklıkla kuzey Amerika ve Avrupa’dandır. Diğer ülkelerden veriler sınırlıdır. Bu çalışma ile Türkiye’deki SMZL hastalarının hepatit serolojisi durumları ve tedavi yöntemlerinin ortaya konulması amaçlanmıştır.

**Gereç ve Yöntemler:**

Türkiye’de farklı merkezlerden IBM SPSS Statistics for Windows v23 elektronik tablo kullanılarak veriler toplanmıştır. Hepatit B virüs yüzey antijeni (HBsAg), Anti-HBs antikor, Anti-HB kor antijen antikoru (anti-HBcAg), HB virüs yükü, anti-hepatit C virüs (HCV) antikoru, HCV virüs yükü sonuçları analiz edilmiştir.

**Bulgular:**

Yüz dört hastanın verilerine ulaşılmıştır. Hepatit C virüs pozitifliği sadece 1 hastada tespit edilmiştir. Hepatit B virüs yüzey antijeni (HBsAg) pozitifliği %11,2, HBsAg ve/veya hepatit B kor antijen antikoru (anti-HBcAg) pozitifliği %34,2 oranında tespit edilmiştir. Ortanca yaş 60 yıl (35-87) olarak saptanmıştır. Ortanca izlem süresi 21,2 (0,2-212) ay (yaşayan hastalar için 23,2 ay) olarak bulunmuştur. Ortanca genel sağkalım (GS) süresine ulaşılamamıştır. Tahmini 3-yıl ve 10-yıl GS oranları %84,8 ve %68,9 olarak bulunmuştur. İleri yaş, splenektomi yapılmaması, <90x10^3^/μL platelet sayısı, hipoalbuminemi, laktat dehidrogenaz yüksekliği, β_2_-mikroglobulin yüksekliği ve HBsAg pozitifliği sağkalımla ilişkili faktörler olarak bulunmuştur. Çok değişkenli analizde, sadece albumin düşüklüğü istatistiksel olarak anlamlı saptanmıştır.

**Sonuç:**

Bu çalışmanın sonuçlarına göre, toplumumuzda SMZL için hepatit B virüsü olası bir risk faktörü olabilir. Aynı zamanda indirek bir prognostik gösterge olabilir.

## Introduction

Splenic marginal zone lymphoma (SMZL) is a rare B-cell lymphoma. It constitutes less than 2% of lymphoid neoplasms [1]. The majority of patients have an indolent course with median overall survival of about 10 years [2,3].

Chronic hepatitis C is frequently observed in SMZL patients. However, these reports are largely from North America and Europe [4,5]. Data from various countries with different hepatitis prevalence rates are lacking.

Many prognostic factors have been described for SMZL, such as leukocytosis, thrombocytopenia, elevated β_2_-microglobulin, anemia, elevated lactate dehydrogenase (LDH), decreased albumin, impaired performance status, advanced age, bone marrow involvement, and histologic transformation [6,7,8,9,10]. Various clinical prognostic scores have been described, but no universally accepted risk stratification formula has been identified.

No curative treatment has been described for this indolent neoplastic disorder. Treatment is indicated in the case of symptomatic disease and/or significant cytopenia. Splenectomy, rituximab, rituximab plus single-agent or multiagent chemotherapy regimens, and recently ibrutinib and idelalisib have been reported to give high treatment success rates [11]. In this multicenter cohort study we aimed to identify the clinical characteristics of SMZL patients in Turkey including viral hepatitis status, treatment details, and survival.

## Materials and Methods

Data were gathered from voluntarily participating centers from different regions of Turkey using IBM SPSS Statistics 23 for Windows (IBM Corp., Armonk, NY, USA). The diagnosis of SMZL, established by the local hematopathologist, was accepted. Diagnoses were based on widening of the white pulp without predominant red pulp involvement and a wide immunohistochemical panel that helped rule out other low-grade B-cell lymphomas and clinicopathologic correlation. The neoplastic B-cell population was immunophenotypically required to lack cyclin D1, CD10, Bcl-6, CD123, annexin-1, and co-expression of CD5 and CD23. A central review in our department of pathology was not obligatory, but statistical evaluations were repeated in the group of cases (n=40) diagnosed at the primary research center, Hacettepe University’s Faculty of Medicine (HUFM). In the case of atypical clinical presentation (e.g., presence of prominent lymphadenopathies in addition to splenomegaly), unexpected morphological, and/or immunophenotypic findings, the submitting center was contacted to confirm the diagnosis. As presented in Table 1, the following data were recorded: age; sex; main reasons for admission to the hospital; leukocyte, lymphocyte, and neutrophil counts and hemoglobin level, platelet count, serum albumin, and β_2_-microglobulin at diagnosis; CD5, CD10, CD20, CD23, CD7, CD103, surface Ig, cyclin D1, and FMC7 results (immunohistochemical or flow cytometry); spleen size; bone marrow involvement; extranodal involvement site; ECOG performance status; and hepatitis B virus surface antigen (HBsAg), anti-HBs antibody, anti-HB core antigen antibody (anti-HBc), HB viral load, anti-hepatitis C virus (HCV) antibody, and HCV viral load results. In addition, the first treatment choice (watch-and-wait, splenectomy, chemoimmunotherapy, etc.), treatment response, and survival status were recorded. Treatment responses were defined as previously reported [12]: 1) hematological improvement (after splenectomy): at least 50% improvement in blood counts; 2) partial response: ≥50% improvement in spleen size, cytopenias, and lymphadenopathies if present, and decrease in the level of marrow lymphoid infiltration; 3) complete response: resolution of organomegaly, normalization of blood counts (hemoglobin >12 g/dL, platelet count >100x10^3^/µL, neutrophils >1.5x10^3^/µL), no evidence of circulating clonal B cells, and no or minor BM infiltration detected by immunohistochemistry; 4) no response or progressive disease: less than partial response or disease progression.

### Statistical Analysis

Categorical and continuous data were expressed as ratio (%) and median (range) and they were compared by chi-square and independent samples t-tests, respectively. Survival analyses were computed by the Kaplan-Meier method. Overall survival (OS) was calculated from presentation to the date of mortality due to any reason. Patients who had not died at the last follow-up were censored at that time. Parameters related to survival were investigated by Cox regression univariate and multivariate analyses. All 7 parameters in Table 2 were included in the multivariable model. All patients gave informed consent for their treatment and information analyses. This study complied with the Declaration of Helsinki. IBM SPSS Statistics 23 for Windows was used for statistical analyses. Values of p<0.05 were considered statistically significant.

## Results

A total of 104 patients, diagnosed between June 1999 and November 2017, were reported from 23 hematology/oncology centers. Forty-seven (45%) of these were diagnosed/confirmed at our center. Data on baseline clinical characteristics are presented in Table 1. The median age was 60 years (range=35-87), and 62.5% of the patients were female. Cytopenia(s) and/or related symptoms (26.8%) and abdominal discomfort (45.4%) were the most frequent reasons for hospital admission. At presentation, 46.1% of patients had B symptoms (fever, night sweats, weight loss), while 8.6% of the patients lacked disease-related symptoms and were diagnosed incidentally. According to ECOG performance scoring, 22.1%, 47.4%, 23.2%, and 7.4% of patients were scored as 0, 1, 2, and 3, respectively. At diagnosis, 77.9% and 49% of patients had bone marrow and peripheral blood involvement, respectively, while 17.3% of patients had prominent lymphadenopathies in addition to splenomegaly.

Eleven of 98 (11.2%) evaluable patients had HBsAg positivity and only 1 of 93 (1.1%) evaluable patients had HCV positivity. Twenty-two of 74 (29.7%) evaluable patients had anti-HBc positivity. The rate of HBsAg and/or anti-HBc positivity was 34.2%. The rate of HBsAg and/or anti-HBc positivity was 30.2% in these cases. The rates of HBsAg and anti-HBc positivities were 13% and 27.9%, respectively, in the cases diagnosed at HUFM. All positive HBV patients received antiviral prophylaxis.

**Figure 1:**
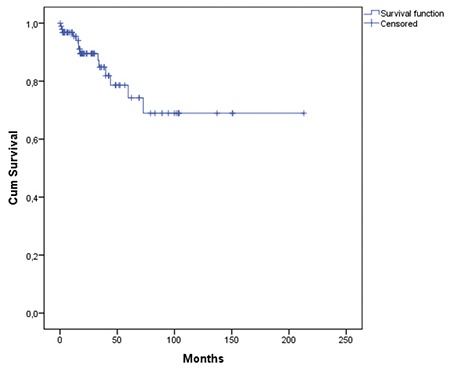
Overall survival of all patients.

Wait-and-watch strategies, splenectomy, and chemo(immune)-therapy were the frontline management methods for 18.4%, 49.5%, and 32.1% of patients, respectively. Only 79 patients were evaluated for response. Hematological improvement and complete response were obtained in the majority of patients (Table 1). Median follow-up duration was 21.2 months (range=0.2-212; 23.2 months for surviving patients). Fourteen (13.4%) patients died during follow-up. Median OS was not reached. Estimated 3-year and 10-year survival rates were 84.8% and 68.9%, respectively (Figure 1).

Older age [hazard ratio (HR), confidence interval (CI): 1.10 (1.03-1.17)], no splenectomy during follow-up [3.88 (1.26-11.88)], platelet counts of <90x10^3^/µL at presentation [3.84 (1.31-11.20)], lower albumin [0.13 (0.03-0.47)], elevated LDH [1.00 (1.00-1.00)], higher β_2_-microglobulin [1.00 (1.00-1.00)], and HBsAg positivity [0.27 (0.08-0.88)] were associated with increased risk of death in the univariate analyses. Only serum albumin level remained marginally significant in multivariate analysis [0.09 (0.00-1.04)]. Univariate and multivariate analyses for survival are shown in Table 2.

## Discussion

In this analysis we report increased prevalence of chronic HBV infection in SMZL patients. HBV exposure is prevalent among adults in Turkey. The reported rate of HBsAg positivity in blood donors was approximately 2%-3% during the last decade [13,14]. In recent epidemiological data, the prevalence was reported as close to 4% [15]. Anti-HCV positivity was reported to be close to 1% in our country [16]. HBsAg was 3.7% and anti-HCV Ab positivity was 2.8% in lymphoma patients in another study from Turkey [17]. We previously reported interim results of this study in 2016 [18]. To the best of our knowledge, we were the first group to suggest a possible association between HBV and SMZL in a considerably large SMZL cohort. Some other studies reported on only a few patients with SMZL associated with HBV [19,20,21,22,23]. Recently, Fetica et al. [24] from Romania found HBV infection in 3 patients out of 34 SMZL patients in the same time period as our early report. A more recent study from China reported HBsAg positivity in 25/160 (16%) and resolved HBV infection (HBsAg negative, anti-HBc positive) in 54/160 (34%) patients [25]. A summary of the data in the literature on HBV and HCV seropositivity is shown in Table 3 [19,20,21,22,23,24,25,26,27,28,29].

Chronic antigenic stimulation is frequently blamed in the pathogenesis of extranodal marginal zone lymphomas. The association between gastric mucosa-associated lymphoid tissue lymphoma and chronic *Helicobacter pylori *infection is the classical example for this relationship. An association between HCV and SMZL has been previously reported in some geographic regions, mostly in South Europe [2,5,29]. Now we can suggest that the association between SMZL and chronic viral hepatitis is not specific for HCV. HBV may also be involved in SMZL lymphomagenesis.

**Figure 2:**
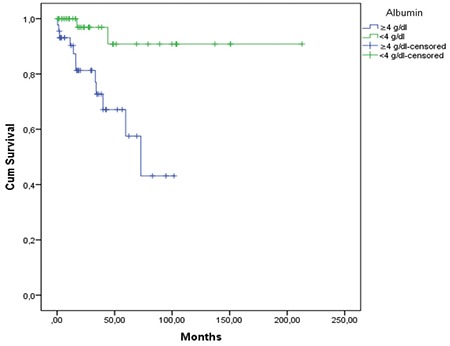
Overall survival according to serum albumin level at diagnosis.

Splenectomy and rituximab-based chemoimmunotherapies were the most frequently used treatments in our cohort. This is in concordance with current treatment strategies for SMZL. Responses (most commonly hematological improvement after splenectomy as expected) were very frequent (94.9%) in our cohort. The median follow-up duration (21.2 months) in our patients was relatively short for this indolent lymphoma. Estimated 10-year survival was 68.6%. We found many parameters (lower albumin, splenectomy, thrombocytopenia, elevated LDH, higher b_2_-microglobulin, and HBsAg positivity) to be associated with overall survival, but albumin was the only parameter to retain marginal significance in multivariate analysis (Figure 2). HBsAg positivity was an adverse prognostic factor in univariate analysis, but not in the multivariate test. It is possible that HBV may indirectly affect survival by lowering serum albumin levels due to liver impairment. This suggestion should be investigated in further studies.

Arcaini et al. [2] reported 10-year OS as 65% in SMZL. In that study, the authors proposed a prognostic model including hemoglobin of <12 g/dL, elevated LDH, and albumin level of <3.5 g/dL as adverse prognostic factors. In another study, Montalbán et al. [5] developed a continuous model for estimating lymphoma-specific survival including decreased hemoglobin level, lower platelet count, elevated LDH, and extrahilar lymphadenopathy as unfavorable prognostic indicators. In a recent Chinese study [25], the authors also suggested a new prognostic system. Decreased hemoglobin, HBsAg positivity, and complex karyotype were related to decreased survival in that study. We did not intend to develop a prognostic scoring system or to test previously suggested scoring systems in our study, but it is convincing to observe that many of the risk factors we identified in univariate analyses have been previously reported to have prognostic significance in SMZL.

The major limitations of this study are its retrospective design and somewhat limited number of patients.

## Conclusion

Our results in association with some recent literature data indicate that HBV may be a possible risk factor for development of SMZL in some geographical regions, similar to HCV in some Western countries. It may also be an indirect prognostic factor. Larger studies about this rare lymphoma would obviously provide better data and firmer conclusions on this relationship and the prognostic impact of HBV.

## Figures and Tables

**Table 1:**
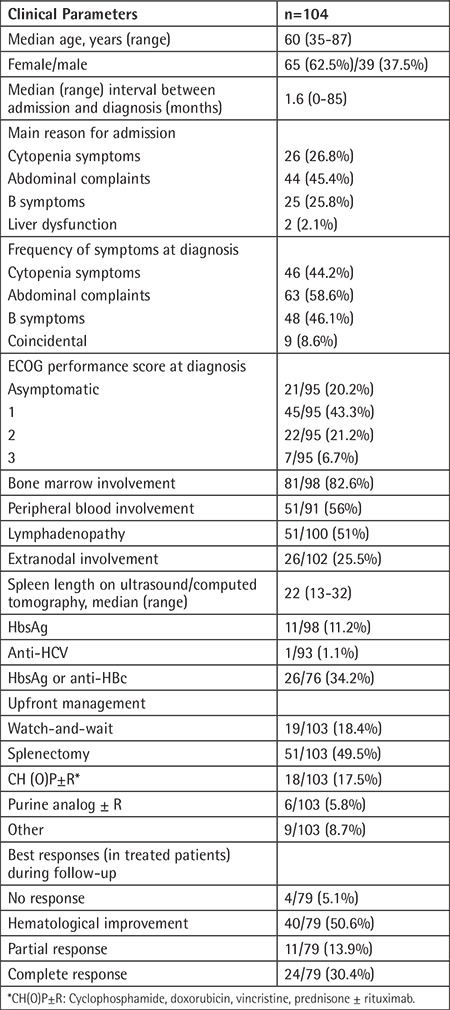
Baseline characteristics and main treatment details of patients.

**Table 2:**
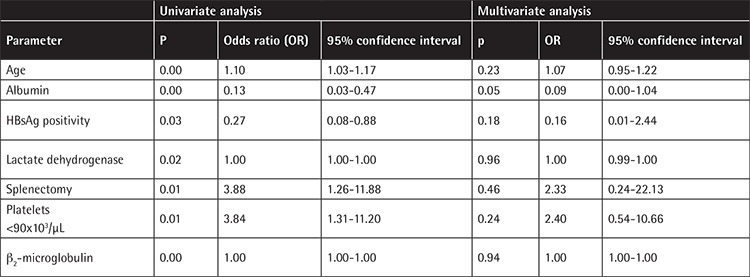
Univariate and multivariate analyses for survival.

**Table 3:**
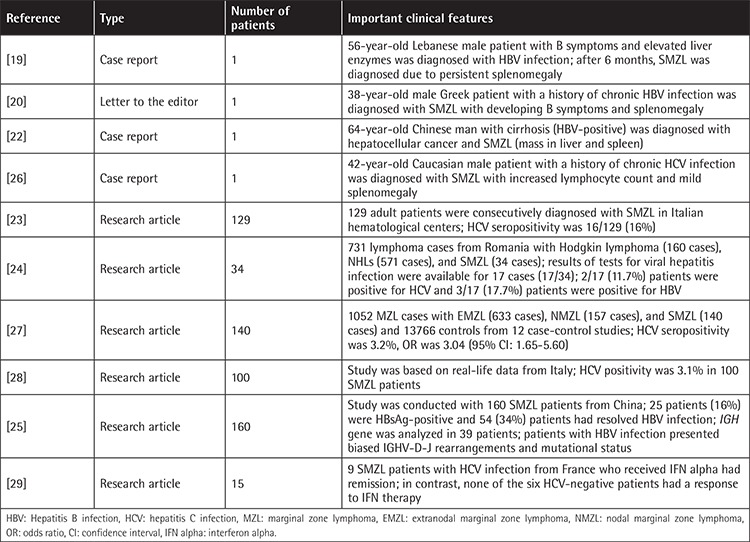
Summary of the data in the literature about hepatitis B and C.
